# Stress and passion predict burnout in regular exercisers

**DOI:** 10.3389/fspor.2026.1781029

**Published:** 2026-06-24

**Authors:** Lili Anna Hujber, Angéla Somogyi, Norbert Tabi, Attila Szabo

**Affiliations:** 1 Doctoral School of Psychology, Faculty of Education and Psychology, ELTE Eötvös Loránd University, Budapest, Hungary; 2 Department of Psychology and Health Management, Faculty of Health and Sport Sciences, Széchenyi István University, Győr, Hungary; 3 Institute of Health Promotion and Sport Sciences, Faculty of Education and Psychology, ELTE Eötvös Loránd University, Budapest, Hungary

**Keywords:** cross-sectional, harmonious passion, obsessive passion, stress, training

## Abstract

**Introduction:**

Athlete burnout is a growing concern in sports psychology. However, the joint associations of stress, harmonious passion, and obsessive passion with burnout outcomes, particularly across burnout dimensions, have received limited attention.

**Methods:**

This cross–sectional study examined how stress, harmonious passion, and obsessive passion predict overall burnout and its three main components using survey data from 345 regular exercisers.

**Results:**

Multiple regression analyses revealed that stress, obsessive passion, and harmonious passion significantly predicted 'exhaustion' (R² = .21): stress and obsessive passion were significant positive predictors (β = .16, *p* = .001; β = .34, *p* < .001), while harmonious passion was a strong negative predictor (β = –.43, *p* < .001). For “reduced sense of accomplishment”, stress and harmonious passion were significant predictors (β = .24, *p* < .001; β = −.34, *p* < .001; R² = .16), whereas obsessive passion showed no unique effect. Regarding “sport devaluation”, all three predictors were significant (R² = .24): stress and obsessive passion were positively related (β = .17, *p* < .001; β = .22, *p* < .001), while harmonious passion was negatively associated with devaluation (β = −.50, *p* < .001).

**Discussion:**

These findings suggest that pressure and passion orientations can significantly influence regular exercisers' susceptibility to burnout. Reducing stress and fostering harmonious passion may help protect regular exercisers from burnout.

## Introduction

Burnout is a negative experience in sport that comprises physiological, emotional, cognitive, and attitudinal elements (1). It is a widely recognized concern among athletes, characterized by chronic emotional and physical exhaustion, a reduced sense of accomplishment, and a devaluation of sport (2, 3). These three components parallel the dimensions of general burnout described by Maslach and Jackson (4). However, the general burnout dimension of depersonalization does not transfer neatly to the sport context. Therefore, research on athlete burnout has reconceptualized it as sport devaluation, reflecting a detached or cynical attitude toward participation in sport (5).

The most apparent sign of athlete burnout is physical and emotional fatigue related to training and competition (6). While athletes and dedicated exercisers often maintain high levels of motivation and commitment, prolonged exposure to stressors can undermine psychological well-being and performance, ultimately leading to burnout. Understanding the psychosocial determinants of athlete burnout has therefore become a central focus in sport psychology research, particularly as burnout has been associated with withdrawal from sport, diminished performance, and long-term mental health effects (7, 8).

Stress has consistently been identified as a strong predictor of athlete burnout [e.g., (9)]. According to cognitive-affective models, chronic stress overwhelms coping resources and accelerates emotional exhaustion and disengagement from sport (10, 11). Empirical studies reveal that exercisers who experience sustained training load, competitive anxiety, or environmental stressors exhibit significantly higher levels of burnout symptoms across all subdomains (12, 13). Because stress is embedded in both competitive sports and exercise environments, identifying psychological factors that may buffer or exacerbate its effects is essential for understanding why some athletes or devoted exercisers thrive under pressure while others deteriorate over time.

Passion for sport, defined within Vallerand's Dualistic Model of Passion, has emerged as a key motivational factor influencing well-being and maladjustment in athletes (14, 15). Harmonious passion reflects an autonomous form of engagement in which sport is integrated into one's identity without internal pressure, supporting balance and healthy functioning. Conversely, obsessive passion reflects a controlled internalization of involvement in sport, leading to rigid persistence and difficulty disengaging (16, 17). This distinction has considerable psychological consequences: harmonious passion is consistently associated with positive outcomes such as flow, satisfaction, and well-being, whereas obsessive passion is related to stress, rumination, and conflict (18, 19).

Research increasingly demonstrates that passion orientations act as important predictors of burnout vulnerability. Athletes driven by obsessive passion are more likely to experience pressure, frustration, and performance-related stress, thereby elevating the risk of burnout (20, 21). In contrast, harmonious passion may protect athletes from burnout by promoting flexible engagement, adaptive coping, and emotional balance (22, 23). Still, relatively few studies have simultaneously examined life stress and both forms of passion in predicting distinct dimensions of burnout within a single comprehensive model in regular exercisers.

One study by Moen et al. (24) investigated how harmonious and obsessive passion, perceived performance, stress, and worry uniquely predicted burnout among young elite Norwegian athletes. Their findings showed that obsessive passion, perceived performance pressures, and negative stress were key contributors to burnout, whereas harmonious passion functioned as a protective factor. Although this work provided valuable insight into how multiple sport-related cognitions and emotions co-occur to influence burnout, the model included a wide array of predictors that may complicate interpretation and limit applicability outside elite youth sport contexts. The current study extends this literature by focusing specifically on the core psychological factors most consistently linked to burnout, stress, harmonious passion, and obsessive passion, within a broader, more ecologically diverse sample of adult regular exercisers. By isolating these key predictors and examining both overall burnout and its subdomains, the present study offers a more streamlined and generalizable understanding of the motivational pathways associated with burnout in regular exercisers.

Although the sport burnout literature has predominantly focused on competitive athletes (2, 7), the present sample of regular exercisers was not far removed from athlete populations in terms of training involvement. Most participants trained at least three times per week, 61.7% reported training in organized settings or with coaching assistance, and nearly one-third had trained for more than 15 years, indicating that the sample was not limited to casual or occasional exercisers. Moreover, participants in organized training settings reported significantly higher exercise intensity, weekly frequency, and training history than self-organized exercisers (see Participants section), suggesting that a substantial portion of the sample may be considered athletes or near-athletes based on their training patterns. Nevertheless, regular exercisers may still differ from competitive athletes in that they are generally less exposed to formal competition and external performance evaluation. At the same time, regular exercise can still become a source of stress and rigid commitment, particularly when participation is internalized in an obsessive rather than harmonious manner (15, 17). Accordingly, the mechanisms linking stress, passion, and burnout may remain relevant in regular exercisers, even if their manifestations differ somewhat from those observed in competitive athletes. Despite growing interest in the relationship between passion, stress, and burnout, several gaps remain in the literature. First, most studies have examined competitive or elite athletes (2, 7), leaving regular exercisers largely underexplored, even though they can experience similar motivational pressures and rigid commitment patterns (17). Second, most prior work has treated burnout as a unitary construct, overlooking the possibility that stress and passion orientations may differentially predict its subcomponents—exhaustion, reduced sense of accomplishment, and sport devaluation. Third, the few studies that did examine multiple predictors together [e.g., (24)] included a wide array of variables, making it difficult to isolate the specific roles of stress and passion. Finally, available evidence is drawn predominantly from Western European and North American samples, with Hungarian exercisers remaining underrepresented.

To address these gaps, the present study aimed to investigate the extent to which stress, harmonious passion, and obsessive passion predict overall burnout among regular exercisers across all ages, as well as its three core subcomponents: emotional/physical exhaustion, reduced sense of accomplishment, and sport devaluation. By adopting a streamlined model focused on the core predictors most consistently linked to burnout and testing them across a demographically diverse sample of regular exercisers (ages 18–78), the current study extends existing knowledge beyond elite sport contexts. Therefore, it may offer a more generalizable understanding of the motivational pathways associated with burnout.

**Figure 1:**
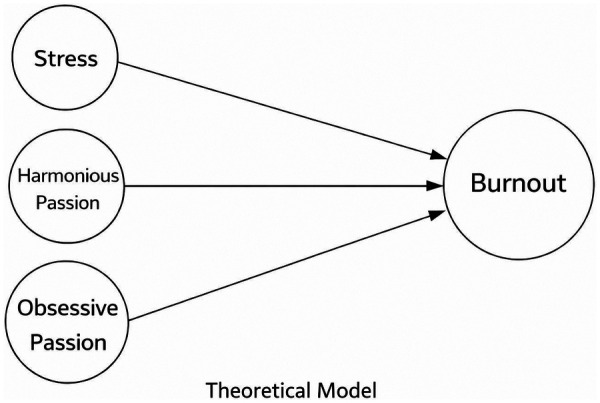
Theoretical model illustrates the tested relationships between stress, harmonious passion, obsessive passion, and burnout in regular exercisers.

Based on the theoretical and empirical evidence reviewed above, three specific hypotheses were formulated, as illustrated in the theoretical model presented in Figure 1. Hypothesis 1 (H1): Drawing on Smith's (11) cognitive-affective model, in which chronic stress depletes coping resources and fosters emotional exhaustion and disengagement, perceived stress was hypothesized to positively predict overall burnout and its subcomponents (exhaustion, reduced sense of accomplishment, and sport devaluation). Hypothesis 2 (H2): According to the Dualistic Model of Passion (15, 17), harmonious passion reflects autonomous internalization that supports flexible engagement and psychological well-being. Therefore, harmonious passion was hypothesized to negatively predict overall burnout and its subcomponents. Hypothesis 3 (H3): Because obsessive passion involves controlled internalization that leads to rigid persistence, internal pressure, and difficulty disengaging from sports and exercise (17), obsessive passion was hypothesized to positively predict overall burnout and its subcomponents. In sum, as depicted in Figure 1, stress and obsessive passion were hypothesized to be agonists of burnout, whereas harmonious passion was hypothesized to be an antagonist. The magnitude of these relationships was exploratory.

## Methods

### Participants

We recruited participants through social media and sport clubs and used snowball sampling to identify individuals who met the study criteria (training at least 3 times per week). The final sample consisted of 345 adults, of whom 168 were men and 177 were women. The mean age of the participants was 25.76 years (SD = 12.17), ranging from 18 to 78 years. All the participants were Hungarians. The intensity of physical activity averaged 7.7 on a self-reported 1–10 scale. Most respondents (40.6%) trained 3 times a week, and 61.7% trained through a sports club or with a coach, rather than individually. Nearly one-third of the sample (112 participants) had trained for more than 15 years. Independent-samples *t*-tests revealed that participants in organized training settings reported significantly higher exercise intensity [*t*(343) = 2.38, *p* = .018, *d* = 0.26], weekly training frequency [*t*(326.70) = 2.98, *p* = .005, *d* = 0.34], and longer training histories [*t*(208.07) = 4.59, *p* < .001, *d* = 0.55] than self-organized exercisers. These findings suggest that the sample comprised a continuum from dedicated regular exercisers to persons whose training patterns closely resemble those of competitive athletes, supporting the feasibility of using the Athlete Burnout Questionnaire in this context.

### Ethics statement

This cross-sectional study was performed with approval (Permission No. SZE/ETT-9/2025 (iii. 13.) from the Scientific Advisory Board's Ethics Committee of the Széchenyi István University. Moreover, the research conformed to the ethical guidelines set out in the British Psychological Society (BPS) Code of Human Research Ethics (25), and the protocol strictly adhered to the research principles for the protection of human participants outlined in the Declaration of Helsinki (26). All participants consented to taking part in the study and to the publication of anonymous group results. Consent was obtained by clicking a “Yes, I agree” button; otherwise, the survey could not be accessed.

### Measures

The survey consisted of several demographic questions, including age, gender, training intensity, years of training, training organization, and weekly training hours. In addition to the demographic and sport activity-related questions, the following psychometric instruments were used to measure passion, stress, and burnout related to sport.

#### Passion—passion scale

Passion was assessed using the Passion Scale Marsh et al. (28), which measures harmonious passion (HP) and obsessive passion (OP). In the present study, the validated Hungarian version of the instrument was applied (27). The scale consists of two 6-item subscales assessing HP (e.g., “Sport/training is in harmony with the other activities in my life”) and two 6-item subscales assessing OP (e.g., “If I could, I would only do sport/training”). Respondents rated the statements on a 7-point Likert scale ranging from 1 to 7. Both the English and the Hungarian versions of the scale demonstrated good psychometric properties. In the original study, the reliability indices (Cronbach's *α*) were 0.83 for HP and 0.86 for OP (28), while in the Hungarian adaptation, *α* values ranged from 0.82 to 0.86 for HP and from 0.82 to 0.85 for OP (27). In the *present sample, Cronbach's α values were 0.83 for HP and 0.85 for OP*.

#### Stress—perceived stress scale

Perceived stress was measured using the Hungarian version of the 4-item Perceived Stress Scale (PSS-4; (29, 30). The scale evaluates individuals' perceptions of stress with items such as: “In the last month, how often have you felt difficulties were piling up so high that you could not overcome them?” Respondents rate the answers on a 5-point Likert scale ranging from 0 to 4, with higher scores indicating greater perceived stress. The PSS-4 assesses general perceived stress rather than sport-specific stress. This choice was deliberate, as the sample included regular exercisers for whom domain-specific sport-related stress measures designed for competitive athletes may be less applicable. Moreover, general life stress has been shown to spill over into the sport context and contribute to athlete burnout (10). In the original version, the internal reliability indicators (*α*) ranged from 0.84 to 0.86 (29), while the Hungarian adaptation showed *α* values between 0.79 and 0.88 (30). In the present study, the internal reliability of the scale was 0.77.

#### Burnout—athlete burnout questionnaire

The level of burnout related to sport activity was measured using the Athlete Burnout Questionnaire [ABQ; (3)]. In the present study, the Hungarian version was applied (31). Consistent with the conceptual model of athlete burnout, the questionnaire has three dimensions: emotional/physical exhaustion (EXH: perceived depletion of physical and emotional resources resulting from sport training and competition), reduced sense of accomplishment (RSA: sense of low accomplishment and personal inadequacy in sport) and sport devaluation (SD: development of a diminished and cynical view towards the benefits of sport participation) (3). Each subscale consists of five items, measuring reduced sense of accomplishment (e.g., “I am not achieving much in sport”), emotional and physical exhaustion (e.g., “I am exhausted by the mental and physical demands of sport”), and sport devaluation (e.g., “I am not into sport like I used to be”). Respondents rate the answers on a 5-point Likert scale ranging from 1 to 5. In the original version, the internal reliability indicators (α) ranged from 0.78 to 0.89 (3), while the Hungarian adaptation showed *α* values between 0.76 and 0.86 (31). In the present study, the internal reliability of the subscales was SD: 0.71, EXH: 0.86, and RSA: 0.78.

### Procedure

The study was conducted via an anonymous online questionnaire in Qualtrics from April to October 2025. The research was anonymous and cross-sectional. Only individuals aged 18 years and older who reported training at least 3 times per week were eligible to participate in the study. It took approximately 10–15 min to complete the questionnaire. Following data collection, the responses were exported to an SPSS file, and the statistical analyses were conducted in IBM SPSS Statistics (Version 30).

### Sample size calculation

*A priori* power analysis was performed using G*Power 3.1.9.7 (32) to determine the minimum sample size needed for the multiple regression models with three predictors: stress, harmonious passion, and obsessive passion. Using a medium effect size (*f*² = 0.15), α = .05, and a power of .95, the analysis indicated that at least 119 participants were required to reliably detect significant effects. The current study greatly exceeded this threshold, with a final sample of 345 exercisers.

### Statistical analysis

All analyses were conducted using IBM SPSS Statistics (V. 30). Normality was tested through skewness and kurtosis tests. Pearson correlations were computed to examine the associations among burnout, its subdomains, stress, harmonious passion, and obsessive passion. Subsequently, multiple regression analyses were performed to test whether stress, harmonious passion, and obsessive passion predicted overall burnout and each burnout subdomain. Assumptions of linearity, homoscedasticity, and multicollinearity were evaluated and met; all VIF values were below 1.20 (38). Statistical significance was set at α = .05.

## Results

### Data normality

Skewness and kurtosis values indicated that the psychological variables were approximately normally distributed, with only mild deviations (skewness < 0.60, kurtosis between −0.60 and −0.20). Such deviations are well within acceptable limits for multiple regression analyses, as they are generally considered sufficient to assume approximate normality in psychological data (33).

### Validity and common method bias tests

Convergent validity was supported by composite reliability (CR) values ranging from .813 (SD) to .903 (EXH), all exceeding the .70 threshold, and AVE values ranging from .472 (SD) to .651 (EXH). While the SD subscale fell marginally below the .50 AVE benchmark, its CR exceeded .70, which is considered acceptable (34). Discriminant validity was confirmed through both the Fornell–Larcker criterion, where the square root of AVE for each construct exceeded its correlations with all other constructs, and the Heterotrait-Monotrait ratio of correlation (HTMT), with all values below the.85 threshold [highest ratio = .694; (35)]. Common method bias was assessed using both Harman's single-factor test and the Common Latent Factor (CLF) technique. The single unrotated factor explained only 24.54% of total variance, and the CLF analysis yielded an average common method variance of 21.45%, both well below the 50% threshold (36, 37). These results indicate that common method bias did not pose a significant threat to the validity of the findings.

### Correlations

The correlation analysis revealed the expected pattern among the study variables (Table 1). Burnout correlated positively with its three subdomains and was negatively related to harmonious passion. Stress showed small but statistically significant positive correlations with overall burnout and two of its components. Obsessive passion demonstrated a small positive association with exhaustion and a small negative association with a reduced sense of accomplishment. However, its relationship with overall burnout was minimal. Harmonious passion, on the other hand, exhibited consistent moderate negative correlations with all burnout indices. Overall, the correlational pattern supports the conceptual distinctiveness of the burnout subdomains and the relevance of stress and passion orientations for subsequent regression analyses.

**Table 1:** Correlation matrix for study variables.

Variable	1	2	3	4	5	6	7
1. Burnout	—	.863***	.722***	.817***	.207***	−.418***	.059
2. BSDV		.—	.677***	.586***	.156**	−.418***	.052
3. BRSA			—	.395***	.173***	−.368***	−.005
4. BEXH				—	.130*	−.305***	.194***
5. Stress					—	.029	.028
6. HP						—	.343***
7. OP							—

BSDV, burnout sport devaluation; BRSA, burnout reduced sense of accomplishment; BEXH, burnout exhaustion; HP, harmonious passion; OP, obsessive passion. Degrees of freedom (df) = 343 for correlations among Burnout, BSDV, BRSA, BEXH, Stress; df = 326 for correlations involving HP and OP. Significance levels: *p < .05, **p < .01, ***p < .001.

Given that approximately 38% of respondents were individual exercisers while the remainder participated in organized sports/exercise, we examined whether the correlation patterns differed between these groups before pooling the data for regression analyses. Specifically, we compared the magnitude of correlations between burnout (and its three dimensions) and stress, OP, and HP across the groups using Fisher's r-to-z transformations. None of the correlations differ significantly between participants in individual and organized sports/exercise (Table 2). This equivalence across groups justified combining samples for subsequent regression analyses.

**Table 2:** Fisher's r-to-z transformation comparisons between two independent samples.

Variable	*r*₁	*r*₂	*z*	*p*
Stress-Burnout	.272	.092	1.670	.095
Stress-BSDV	.212	.052	1.459	.145
Stress-BRSA	.209	.106	0.945	.345
Stress-BEXH	.147	.136	0.100	.920
OP-Burnout	.039	.025	0.125	.900
OP-BSDV	.032	.010	0.197	.844
OP-BRSA	.008	−.059	0.600	.549
OP-BEXH	.186	.124	0.568	.570
HP-Burnout	−.407	−.492	0.954	.340
HP-BSDV	−.402	−.517	1.307	.191
HP-BRSA	−.328	−.462	1.424	.155
HP-BEXH	−.310	−.369	0.596	.551

r₁ = correlation in Sample 1 (*n* = 213); r₂ = correlation in Sample 2 (*n* = 132). BSDV, burnout sport devaluation; BRSA, burnout reduced sense of accomplishment; BEXH, burnout exhaustion; OP, obsessive passion; HP, harmonious passion. All comparisons were non-significant at the *p* < .05 level.

### Regression analyses

A multiple regression analysis explored whether stress, HP, and OP predicted overall burnout. The model was statistically significant, *F*(3, 324) = 40.75, *p* < .001, and explained 26.7% of the variance in burnout (adjusted *R*^2^ = .267). Stress was a significant positive predictor (β = .20, *p* = .001), indicating that regular exercisers who experienced higher stress also reported greater burnout. HP was a strong negative predictor, β = –.50, *p* < .001, suggesting that higher HP was associated with substantially lower burnout. OP was also a significant positive predictor, β = .23, *p* < .001, indicating that regular exercisers with greater OP reported higher burnout. Collinearity diagnostics showed no concern (all VIFs < 1.20), and no influential cases were identified. Next, we examined further how the three predictors affected the three burnout subdomains: physical/emotional exhaustion, sense of accomplishment, and sport devaluation.

The first follow-up regression analysis investigated whether stress, HP, and OP predicted emotional and physical exhaustion. The overall model was statistically significant, *F*(3, 324) = 30.45, *p* < .001, and explained 21.3% of the variance in exhaustion (adjusted *R*^2^ = .213). As shown by the standardized coefficient, HP was a strong negative predictor of exhaustion, β = –.43, *p* < .001, indicating that regular exercisers with higher HP experienced notably lower levels of exhaustion. Conversely, OP was a significant positive predictor, β = .34, *p* < .001, indicating that higher OP was associated with greater exhaustion. Stress also had a modest contribution to the model, β = .16, *p* = .001, with increased stress predicting greater exhaustion. Collinearity diagnostics showed no concern (all VIFs < 1.20), and no influential cases were identified.

The second regression analysis examined whether stress, HP, and OP predicted a lower sense of accomplishment. The overall model was statistically significant, *F*(3, 324) = 24.72, *p* < .001, explaining 16.2% of the variance in reduced accomplishment (adjusted *R*^2^ = .162). Stress was a significant positive predictor, β = .24, *p* < .001, indicating that regular exercisers with higher stress also reported greater reduced accomplishment. HP was a significant negative predictor, β = –.34, *p* < .001, indicating that regular exercisers with higher HP experienced less reduced accomplishment. OP, however, did not significantly predict the reduced sense of accomplishment, β = –.003, *p* = .951. Collinearity statistics showed no issues (all VIFs < 1.20). One case had a high standardized residual, but Cook's distance indicated that it was not influential.

**Figure 2:**
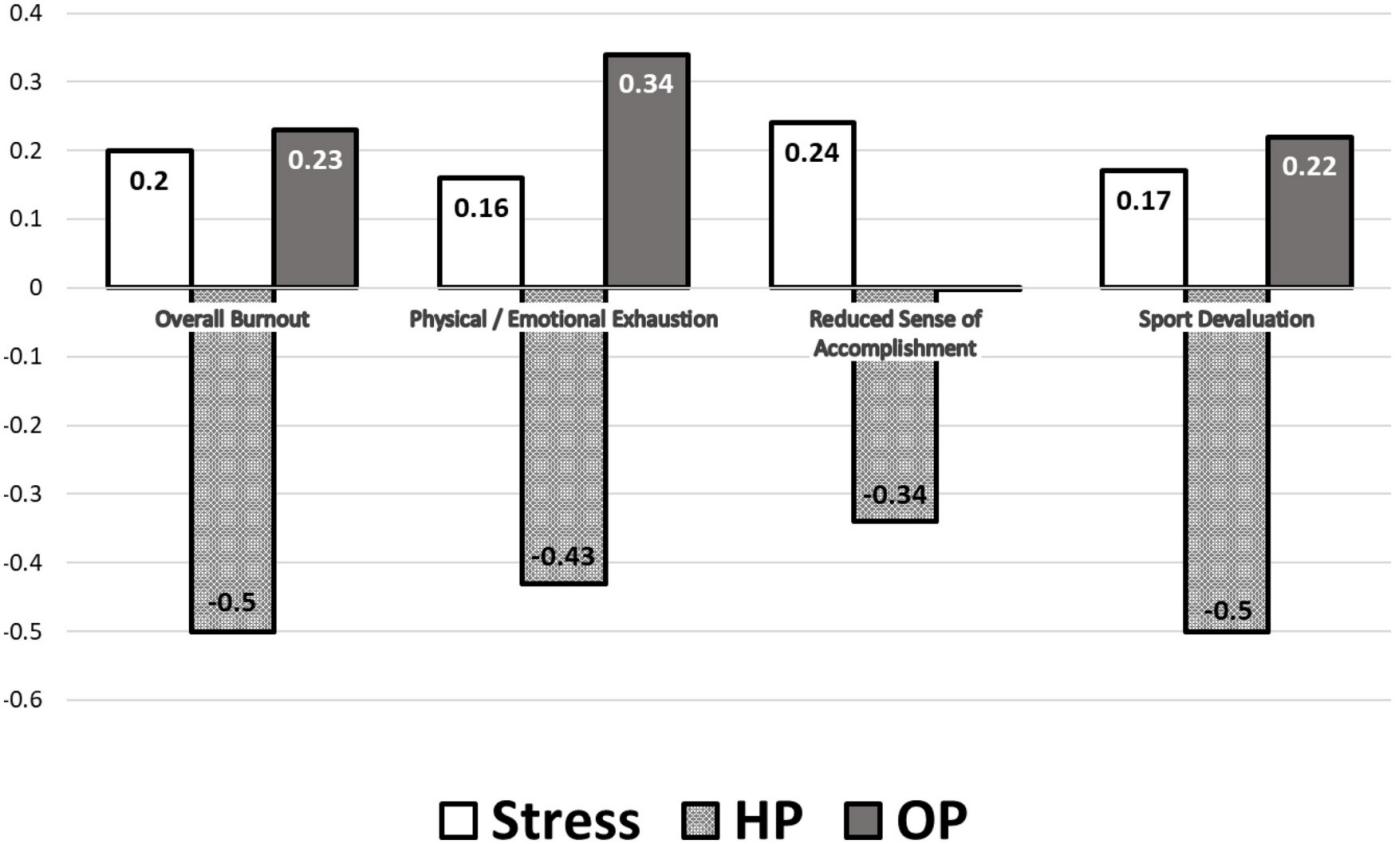
Standardized regression coefficients (β) for stress, harmonious passion (HP), and obsessive passion (OP) predicting overall burnout in regular exercisers and its three subdimensions, illustrating harmonious passion as a consistent protective factor and stress and obsessive passion as risk factors. (HP, harmonious passion; OP, obsessive passion).

Finally, a third follow-up multiple regression analysis was performed to see if stress, HP, and OP predicted sport devaluation. The overall model was statistically significant again, *F*(3, 324) = 35.27, *p* < .001, explaining 24.6% of the variance in devaluation (adjusted *R*^2^ = .239). Stress was a significant positive predictor, β = .17, *p* < .001, meaning more stressed regular exercisers were more likely to feel detached from or less interested in their sport. HP was a strong negative predictor, β = –.50, *p* < .001, indicating that higher HP was associated with lower devaluation. OP was also a significant positive predictor, β = .22, *p* < .001, indicating that greater OP was associated with greater sport devaluation. Collinearity diagnostics revealed no issues (all VIFs < 1.20), and although two cases had high standardized residuals, Cook's distance showed neither was unduly influential. Supplementary hierarchical regression analyses controlling for gender and age confirmed that all predictor effects remained significant and virtually unchanged in magnitude across all four models. The results of the regressions are summarized in Figure 2.

## Discussion

The purpose of the present study was to examine the role of perceived stress and passion in predicting overall athlete burnout and its three components separately in Hungarian regular exercisers. The results showed that passion orientation, specifically higher HP and lower OP, is a key factor influencing overall burnout, beyond the effect of stress. These findings support the Dualistic Model of Passion (15), demonstrating that the two forms of passion have distinct effects. While the associations between stress, passion, and burnout have been examined in prior research, the present study offers several new insights. First, unlike most previous work that focused on elite or competitive athletes (2, 7), the current study targeted regular exercisers, a population that has been underrepresented in the burnout literature despite experiencing similar motivational pressures. Second, by examining the three burnout subdimensions separately, the study revealed that OP's role is not uniform across burnout components—it was unrelated to reduced sense of accomplishment but positively associated with exhaustion and sport devaluation—a pattern not previously documented in non-elite samples. Third, the streamlined three-predictor model isolates the core psychological factors most consistently linked to burnout, providing a clearer and more parsimonious test of the theoretical framework compared to studies that included a wide array of predictors [e.g., (24)]. Finally, the study extends the evidence base to a Hungarian sample, addressing the cultural underrepresentation in this area of research.

Regarding the stated hypotheses, H1 was fully supported: perceived stress was a significant positive predictor of overall burnout and all three subcomponents (exhaustion, reduced sense of accomplishment, and sport devaluation). H2 was also fully supported: harmonious passion emerged as a significant negative predictor of overall burnout and each of its subdimensions, confirming its hypothesized protective role. H3 was partially supported: obsessive passion was a significant positive predictor of overall burnout, exhaustion, and sport devaluation, but it did not significantly predict reduced sense of accomplishment (β = –.003, *p* = .951). Thus, the theoretical model depicted in Figure 1 was largely confirmed, with the notable exception that obsessive passion does not appear to uniformly contribute to all facets of burnout.

These findings are partially consistent with previous research and extend earlier results. Moen et al. (24) found that HP, perceived performance, stress, and worry explained athlete burnout, while OP was unrelated to burnout. Similarly, a recent study involving NCAA Division I athletes reported that only HP showed the expected association with athlete burnout, whereas OP did not directly predict burnout (23). These differences between the present study and those reported in NCAA Division I athletes (23) and in junior elite athletes (24) may partly be explained by differences in the sport context. The NCAA Division I athletes and junior elite athletes are characterized by intensive training demands and strong commitment to performance. In such settings, OP may reflect adaptive persistence. In contrast, the present sample included recreational and non-elite adult regular exercisers as well. In this context, OP may be more likely to manifest rigid engagement, increasing the vulnerability to burnout. These findings highlight that the role of OP in athlete burnout may also depend on the competitive level and institutional structure of the sporting environment. According to our findings, both HP and OP should be considered when examining athlete burnout. Overall, HP was the strongest (protective) inverse predictor of athlete burnout, supporting previous research.

Besides passion, perceived stress was also a key factor regarding athlete burnout. Higher stress was associated with higher levels of burnout, consistent with the cognitive-affective model of burnout that suggests that chronic stress overwhelms coping resources and accelerates emotional exhaustion and disengagement from sport (11).

The results also suggest that passion orientation, specifically higher HP and OP, seemingly plays a key role in influencing athletes' emotional and physical exhaustion aspects of burnout beyond the effects of stress. OP and stress were significant positive predictors, while HP was a strong negative predictor. These findings align with previous research showing that OP is linked to overinvestment, difficulty disengaging, and sustained effort when fatigued, which may lead to exhaustion (21, 22). In contrast, HP appears to be a protective factor against emotional and physical exhaustion by promoting flexible engagement and adaptive recovery.

Furthermore, the results suggest that both stress and HP substantially contribute to the perceived reduction in accomplishment, with higher stress associated with a greater perceived reduction. In contrast, HP was negatively related to this dimension. According to the meta-analysis by Lin et al. (9), the association between HP and reduced sense of accomplishment is robust, and the present findings also support this. In contrast, OP does not seem to play a unique role in this burnout dimension, making reduced sense of accomplishment the only component in which OP was not a significant predictor. This result suggests that regular exercisers with higher OP may still experience a sense of achievement or competence, even when they report emotional and physical exhaustion and devaluation of sport (as explained in the following paragraph). These findings differ in part from those summarized by Bicalho & Da Costa (20), who reported a positive association between OP and reduced personal accomplishment in elite athlete samples. This discrepancy further supports the fact that the role of OP may vary across competitive levels. Overall, the present findings indicate that while higher stress undermines feelings of effectiveness and progress, HP may strengthen regular exercisers' sense of competence and accomplishment.

Finally, both passion types, especially HP, may be key factors in the sport devaluation aspect of burnout among regular exercisers, beyond the effects of stress. Consistent with Dallman et al. (23), HP was strongly and negatively associated with sport devaluation, indicating a protective effect. However, unlike their findings, the present study showed a positive association between sport devaluation and OP. Although Bicalho & Da Costa's (20) systematic review consistently identified HP as a protective factor against sport devaluation, findings regarding OP were mixed across studies, with variability depending on sample characteristics and sport context. In summary, the present results suggest that obsessive passion may contribute to cynical attitudes toward participation in sport, particularly in recreational and non-elite settings. Perceived stress and OP may contribute to devaluation, whereas the association between HP and devaluation is strongly negative.

In summary, HP appears to play a consistently protective role across overall burnout and all burnout dimensions separately. In contrast, the role of OP remains less clear and may be context-dependent, particularly at lower levels of accomplishment and when sport is devalued. In the present study, OP was unrelated to reduced sense of accomplishment but was positively associated with emotional and physical exhaustion and sport devaluation, suggesting that its effects may differ between elite and non-elite sport contexts. Future research should further examine when OP functions as a risk or neutral factor in athlete burnout.

It is important to note that the explained variance in burnout and its subcomponents was modest, ranging from 16% to 27%. These values are comparable to those expected from prior meta-analytic evidence. For example, Lin et al. (9) reported an overall correlation between athlete stress and burnout of r = .505, corresponding to approximately 25% shared variance. Although this bivariate estimate is not directly equivalent to the multivariable *R²* values in the present study, it suggests that the variance explained by the full model falls within a plausible range for athlete-burnout research. These findings also indicate that a substantial proportion of the variance in burnout remains unexplained by the three predictors examined. This suggests that additional factors, such as social support, coping strategies, training load, and coaching behavior, likely contribute to burnout and its subcomponents. Therefore, the present findings should be interpreted with appropriate caution, and the practical significance of the observed associations should not be overstated.

### Limitations

The study has several limitations to consider. First, the cross-sectional design does not allow causal conclusions about the relationships among stress, passion, and burnout. Second, the variables were assessed using only self-report measures, which may introduce response bias. Third, the sample comprised exclusively Hungarian respondents; thus, the findings may not be generalizable to regular exercisers from other cultural contexts. Fourth, the types of sports were not explicitly examined; consequently, differences between individual and team sports could not be explored. Further research should examine the types of sports in terms of passion, stress, and burnout. Fifth, amateur and professional regular exercisers were not separated, as the only criterion was participation in sport at least 3 times per week. Sixth, although the sample spanned a wide age range (18–78 years), the distribution was strongly skewed toward younger adults (74.8% aged 18–23, M = 25.76, SD = 12.17). Consequently, the findings may not generalize equally across the lifespan. Future research could examine whether the relationships between stress, passion, and burnout differ across age groups, particularly given that exercise motives and contextual demands may shift with age. Seventh, stress was assessed using the PSS-4, which captures general perceived stress rather than sport-specific stress. This creates a conceptual mismatch between the predictor (general stress) and the outcome (sport-specific burnout), which may constrain the interpretation of stress as a direct determinant of sport burnout. Although general life stress has been shown to spill over into the sport domain (10), the PSS-4 may not fully capture sport-specific stressors directly linked to burnout. Future studies could complement this measure with sport-specific stress instruments to disentangle the relative contributions of general and sports or exercise-related stressors to burnout. Eighth, the present study relied on multiple regression analysis, which, while methodologically sound, does not test interaction or mediation effects. Given the theoretical framework, it is plausible that passion orientations may moderate the relationship between stress and burnout (e.g., HP buffering the effects of stress) or mediate motivational processes underlying burnout. Future research should employ moderation and mediation analyses to test these mechanistic pathways. Ninth, the use of multiple regression rather than structural equation modeling (SEM) represents a further analytical limitation. Because all constructs were measured using multi-item scales, SEM would allow simultaneous estimation of measurement error and structural paths, providing a more robust test of the theoretical model. Future studies should consider employing SEM or latent variable modeling to better capture the complexity of the relationships among stress, passion, and burnout (14).

Taken together, these limitations suggest that the present findings are most directly applicable to young adult regular exercisers with high training commitment rather than to competitive athletes or exercisers across the full lifespan. It should also be noted that, although terms such as “predictor” and “protective factor” are used throughout the manuscript in a statistical sense (i.e., reflecting regression-based associations), the cross-sectional design precludes causal inferences. These terms should be interpreted as indicating the direction and strength of associations rather than establishing causality. Future research should aim to replicate the current findings in more diverse cultural and sporting settings. Longitudinal designs should also be conducted in the future to examine the causal relationships among stress, passion, and burnout. In addition, further studies should also explore effective intervention strategies for regular exercisers at risk of burnout.

## Conclusions

This study makes a valuable contribution to the sport-related burnout literature by showing that passion orientation and perceived stress together influence burnout outcomes. In line with the Dualistic Model of Passion, HP emerged as a strong negative predictor of burnout. Conversely, OP and perceived stress were significant positive predictors of athlete burnout. Importantly, these findings highlight that passion and stress should not be studied in isolation but as interacting psychological processes that affect regular exercisers' well-being. Additionally, the distinct patterns across the three burnout dimensions provide clear evidence that burnout is not a single construct. Its components—such as emotional and physical exhaustion, reduced sense of achievement, and sport devaluation—are differently impacted by passion orientation and stress. This underscores the need for dimension-specific analysis when examining burnout in sports. From a practical standpoint, the results have direct implications for trainers, coaches, and sport psychology practices. If confirmed by longitudinal research, preventing burnout may require more than generic stress-management techniques; it also requires actively developing HP among regular exercisers. By promoting autonomous engagement, enjoyment, and flexible involvement in sport, practitioners may help foster resilience to burnout and support more sustainable engagement in sport and exercise. Overall, these findings reinforce the importance of passion-centered approaches as a key part of athlete well-being and long-term performance.

## Data Availability

The raw data can be found in the Supplementary Material. The data were deposited on Mendeley Data repository: https://data.mendeley.com/datasets/hj5jknzww5/1. DOI: 10.17632/hj5jknzww5.1.
